# Discriminable Multi-Label Attribute Selection for Pre-Course Student Performance Prediction

**DOI:** 10.3390/e23101252

**Published:** 2021-09-26

**Authors:** Jie Yang, Shimin Hu, Qichao Wang, Simon Fong

**Affiliations:** 1 Department of Computer and Information Science, University of Macau, Taipa 999078, China; yb77401@um.edu.mo; 2 College of Artificial Intelligence, Chongqing Industry & Trade Polytechnic, Chongqing 408000, China; 3 School of International Relations, Xi’an International Studies University, Xi’an 710128, China; wangqichao0410@outlook.com; 4 ZIAT DACC Laboratory, Zhuhai Institutes of Advanced Technology of the Chinese Academy of Sciences, Zhuhai 519000, China

**Keywords:** educational data mining, academic early warning system, student performance prediction, multi-label learning, attribute selection

## Abstract

The university curriculum is a systematic and organic study complex with some immediate associated steps; the initial learning of each semester’s course is crucial, and significantly impacts the learning process of subsequent courses and further studies. However, the low teacher–student ratio makes it difficult for teachers to consistently follow up on the detail-oriented learning situation of individual students. The extant learning early warning system is committed to automatically detecting whether students have potential difficulties—or even the risk of failing, or non-pass reports—before starting the course. Previous related research has the following three problems: first of all, it mainly focused on e-learning platforms and relied on online activity data, which was not suitable for traditional teaching scenarios; secondly, most current methods can only proffer predictions when the course is in progress, or even approaching the end; thirdly, few studies have focused on the feature redundancy in these learning data. Aiming at the traditional classroom teaching scenario, this paper transforms the pre-class student performance prediction problem into a multi-label learning model, and uses the attribute reduction method to scientifically streamline the characteristic information of the courses taken and explore the important relationship between the characteristics of the previously learned courses and the attributes of the courses to be taken, in order to detect high-risk students in each course before the course begins. Extensive experiments were conducted on 10 real-world datasets, and the results proved that the proposed approach achieves better performance than most other advanced methods in multi-label classification evaluation metrics.

## 1. Introduction

One of the key indicators of high-level education quality is students’ performance in the setting of the learning environment. Studies have shown that the early learning stage of the course is crucial [1,2,3], in which the students are able to nurture their interests in the relevant learning through the understanding and digestion of the syllabus structure and content organization, forming a solid foundation for the subsequent learning stages [4,5]. Adelman et al. [6] conducted a long-term and systematic statistical study on behalf of the National Center for Education Statistics in the US, in order to reveal the constellational correlation and significance of the class attainment, attendance, curriculum, and student performance with the elucidation of what, when, where, and how they study. However, the teachers’ failure to follow up with the class’ progress, or the incomprehensibility of the learning materials, may cause some students to lose interest in learning or eventually give up, seriously affecting their learning behavior for subsequent courses. In addition, due to the low ratio of teachers to students in university courses, it is a great challenge for the teachers to pay close attention to each student. Thus, teaching management tasks—such as teaching in an individual orientation or early warning of academic dysfunction at the beginning of the course—are particularly necessary.

At present, a great number of student performance and transcript-related data are stored in the relevant information systems of educational institutions, which are often dormant in the data system, without being fully utilized and referenced. With the continuous advancement of artificial intelligence technology, various fields—such as medicine [7,8], manufacturing [9], engineering optimization [10], speech recognition [11], and image processing [12]—have adopted and applied the combination of big data analysis and artificial intelligence algorithms radiating novel, cause-driven vitality [13,14]. With the advancement of education informatization and the promotion of smart campuses, colleges and universities have gradually accumulated massive educational data resources [15]. A compelling need has arisen to extract valuable information from these educational informational data in order to better serve and support education and teaching management. As a new branch of research, educational data mining has drawn more attention of late—especially in the prediction of student performance.

As a key component in the development of an academic early warning system, student performance prediction aims to uncover information from various aspects, such as the learning situation of different courses, including selective and compulsory courses, training courses, and other types of lectures, academic exchanges, etc. Despite extensive research on student performance prediction, the extant approaches still have several major limitations.

First of all, many studies are related to e-learning platforms, which have a considerable reliance on students’ online learning behaviors and activities, which the traditional classroom teaching scenarios may not be able to provide [16]. Secondly, most existing methods can only predict either during [16,17] or near the end of a course [18,19], which is ineffective and inadequate for helping students with early learning issues. Similarly, the subsequent courses are often submodules of a complete course syllabus, or advanced courses; therefore, the predicted content 10 datasets functions as a multiple-course measurement. Multi-course prediction essentially belongs to the multi-label problem. The single-label methods often fail to consider the correlation between labels when dealing with multi-label problems. In reality, not only are the follow-up courses and the previous courses related, the following courses are often coupled as well. Lastly, very few studies have focused on the redundant features in the learning data. For example, some selective courses have limited impact on subsequent professional courses, and even some public courses—such as physical education—may not be relevant to most professional courses, but may have indirect relevance to some majors, such as automobiles or engineering industry design. Therefore, redundant features in student performance data will affect the prediction results.

In order to solve the above-mentioned problems, this paper focuses on constructing a multi-label attribute selection model to predict the performance of pre-class students. As far as we know, this is the first study to use a multi-label attribute selection algorithm based on multi-objective optimization to predict the performance of pre-course students. This paper constructs a multi-label attribute selection algorithm by using interclass recognition and intraclass domain recognition. At the same time, it considers the association between features, labels, and the correlation between features and labels, and improves the expressive ability of features by reducing attributes and enhancing the prediction effect.

The main contributions can be summarized in the following three aspects:

- For the first time, we used the multi-label attribute selection method to transform the pre-class student performance prediction problem into a multi-label learning model, and then applied the attribute reduction method to scientifically streamline the characteristic information of the courses taken, along with mining the characteristics of the previous courses for the upcoming advanced or upper courses. The attributes of the curriculum were significant in studying academic early warning from a new perspective, from pre-class student performance prediction to subsequent courses;
- We perceived the task as a multi-label learning problem, which can fully uncover the correlation between the students’ previous course information and multiple target courses, so as to detect and screen out high-risk students in each course prior to the start of the course;
- We collected a new set of student performance prediction data, and proposed a novel multi-label attribute selection method, which improved the ability to express feature information of the previously completed courses.

The layout of this article is organized as follows: Section 2 conducts a comprehensive literature review of the related work. Section 3 introduces our discriminable pre-course student achievement prediction framework in detail. Experimental results and analysis reports are in Section 4, followed by conclusions and future prospects in Section 5.

## 2. Related Work

Set in the context of the traditional classroom teaching scenario, the existing research on course performance prediction is mainly based on students’ performance in the target course (the course to be predicted) [20], such as attendance, homework completion, periodical exam scores, etc. Since the data are remarkably dependent on information obtained in the process of the target course, modeling work is often carried out during the course [17,20], or even near its end [21]. Marbouti et al. [16] used attendance, tests, and weekly homework five weeks after the beginning of the course to predict whether students were at risk of failing the course. Meier et al. [17] used homework, test scores, and course project completion information to predict students’ final grades four weeks after the course started. Some studies [22,23] considered students’ performance in the midterm exams of the course, leading to the prediction of performance only after halfway through the teaching process.

In sum, the above-mentioned studies have shown significantly severe hysteresis in the prediction of course performance, such that they cannot provide effective support for teaching management at the early stage of the course taught. Sweeney et al. [4] regarded the students as users and the selected courses as commodities from the perspective of the recommendation system, and predicted students’ course grades in the following semester by decomposing and completing the course grade matrix. Although such an approach was able to predict the course performance before its start, it often encountered the problem of cold start, and has high requirements on the number of users (sample volume) as well as being difficult to use for modeling tasks on small-scale data.

As one of the most important and popular topics in educational data mining, student performance prediction has attracted a lot of research attention in recent decades. Due to the convenience of data collection, most existing studies on this topic are related to e-learning platforms, including MOOCs (massive open online courses) [24,25], ITSs (intelligent tutoring systems) [26], LMSs (learning management systems) [27,28,29,30], HOU (the Hellenic Open University) [31,32], and other such platforms [33,34,35]. For example, Ren et al. applied the data in the MOOC server log to predict results such as the average daily learning time, the total hours of video-watching, the number of videos watched by students, and the number of tests t conducted [25]. Conijn et al. [36] explored the associations of different MOOC data—including the frequency of MOOC activities, specific course items, and activities—with learner grades in order to predict student performance and, thus, discover the potential for MOOC improvement. Based on LMS tracking data, Macfadyen and Dawson developed a predictive model for students’ final grades, including the number of discussion messages posted, the number of emails sent, and the number of completed evaluations [29]. Zafra et al. predicted students’ performance (i.e., pass or fail) with the information about quizzes, assignments, and forums stored in Moodle, which is a free learning management system [30]. Oswaldo et al. [37] compared different educational data mining (EDM) algorithms on student data from a private computer science college to assess the effectiveness of educational data in improving decision making, while the core of the analysis was to discover research trends and patterns in graduation rate indicators [38]. It is safe to conclude that the above-mentioned research on e-learning platforms mainly relies on the data of students’ online activities, and these data are virtually inaccessible in traditional classroom teaching scenarios.

Sweeny et al. [4] developed matrix completion methods and used them to predict grades for each student for the next enrollment term based on information on grades that students earned on completed courses. Although this model can predict student performance prior to a course’s commencement, it works from the perspective of recommender systems, and significantly differs from our study.

The work most related to ours is that by Ma et al. [22,39], who used a multi-label multi-instance algorithm to predict pre-class student performance, but during data preprocessing, some curriculum features that were considered irrelevant were forcefully removed, and the correlation among features, and between features and labels, was not fully considered, and some possible relevant course feature information was directly ignored. For example, some selective courses may have a certain effect on subsequent professional ones, and the direct deletion of selective course information may lead to some feature effects that weaken the predictive performance. The recent studies are summarized in Table 1.

## 3. Methods

In this study, we develop a pre-processing solution of attribute selection methods with a multi-label course, with a focus on more realistic course data, combined with a portfolio of classifiers to develop more time-sensitive student performance prediction models. A brief introduction to the basics of multi-label learning is first presented, followed by the basic concepts and model framework of the proposed multi-label attribute selection algorithm. Comparative experiments are then conducted in 10 real course datasets and analyzed accordingly.

### 3.1. Multi-Label Learning

In traditional supervised learning, each sample of the dataset possesses only one category label; this is the single-label problem. For example, the handwritten number recognition task corresponds to only one digital label per image sample. In the diagnosis of benign and malignant tumors, each single datum or group of patient data often corresponds to a tumor label that needs to be diagnosed. However, the real world is complex, and the samples in many tasks are inherently ambiguous [40]. It is quite common that in the classification of news topics, a piece of news is likely to belong to multiple labels—such as sports, entertainment, business, and education—at the same time; similarly, in the course performance prediction problem, the previous recorded performance of a non-final-year student majoring in automation can correspond to the performance prediction of multiple courses in the future (such as process control technology, robot control technology, pattern recognition and intelligent systems, etc.).

From the perspective of modeling methods, the existing multi-label learning methods can generally be divided into the following two categories: (1) Converting multi-label issues into other known issues, such as binary classification, multi-class problems, and ranking problems. Take the multi-label learning algorithm ML-SVM [41] as an example; the algorithm reuses each multi-label sample, and trains the model for each label belonging to the sample. The sample is treated as a positive example during model training, and then based on the idea of one-vs.-all, which is transformed into multiple binary classification problems as well as modeling, and classified by using SVM. (2) Modifying existing algorithms (such as supervised learning algorithms) to make them suitable for handling multi-label problems. Take ML-KNN [42] as an example, which modifies the k-nearest neighbor algorithm to adapt to multi-label scenarios. Given a new sample P, first find the k-nearest neighbor samples of P in the training set, and then count the number of neighbor samples for each category with a final step to estimate the label of P with the maximum a posteriori probability (MAP) method.

### 3.2. Multi-Label Attribute Selection

**Figure 1:**
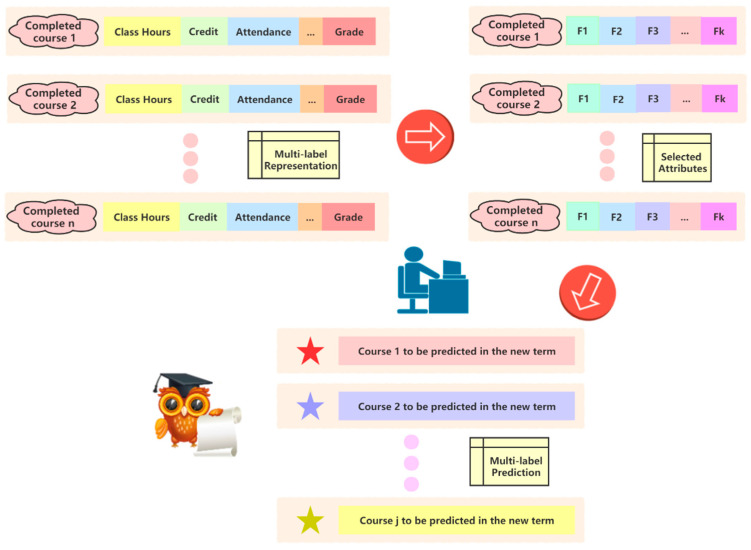
Structure of multi-label attribute selection for pre-class student performance prediction.

In the multi-label feature selection problem, an ideal feature is one that is highly associated with the label. Based on the aforementioned analysis, a certain function may have a higher correlation with a specific label, which at the same time could have a lower relevance with other labels. When it comes to the influence of each feature on each label separately, the multi-label feature selection problem is transformed into a multi-objective optimization problem, and the objective function is regarded as the association between each element and the existing label. In this context, we propose an attribute selection method for the multi-label (AMuL) data processing problem. The basic architecture is shown in Figure 1. Firstly, the course information is feature-extracted to obtain a multidimensional space with a multi-attribute and multi-label structure. Next, all features are distributed into the constructed target space based on the correlation between all features and labels. The next step is attribute selection via a multi-label method, which detects non-dominated solutions (features) in the deterministic space that correspond to the Pareto optimal set in the target space and, finally, performs multi-label prediction.

To find the correlation between attributes and labels, the symmetric uncertainty (SU) indicator [43] is applied. It is common sense that multi-label attribute selection aims to select a set of attributes with the greatest dependence on all classified labels. Therefore, this strategy is called the maximum dependence criterion. The maximum dependency comes from the concepts of entropy and information gain. The entropy can be used to evaluate the uncertainty of a random variable, and one effective method for evaluating random variables is Shannon’s entropy [44]. If we take a discrete random variable *X* whose value belongs to a domain *Vx*, and the probability density function is $p\left(x\right)\;=P\left(X=x\right)$, x∈V the *X* entropy is defined as follows:

(1) $$H\left(X\right)\;=-\overset{n}{\underset{i=1}{\sum }}p\left(X_{i}\right)log_{2}\;p\left(X_{i}\right),\;H\left(X\right)\;\geq 0$$

If *X* and *Y* are two discrete random variables with joint probability density function *p*(*x*,*y*), then the joint entropy of *X* and *Y* is defined. When *X* is known, the conditional entropy is defined as follows:

(2) $$H\left(Y|X\right)\;=-\underset{x\in V_{x}}{\sum }\underset{y\in V_{y}}{\sum }p\left(x,y\right)\log_{2}p\left(x,y\right)$$

The mutual information that can be used to measure the relevance between the two variables *X* and *Y* is defined as follows:

(3) $$H\left(Y|X\right)\;=-\underset{x\in V_{x}}{\sum }p\left(x\right)H\left(Y|X=x\right)\;=-\underset{x\in V_{x}}{\sum }\underset{x\in V_{y}}{\sum }p\left(x,y\right)\log_{2}p(y|x)$$

If the mutual information of *X* and *Y* is large (small), it means that *X* and *Y* are closely (not closely) related. If *I* (*X*,*Y*) = 0, then *X* and *Y* are totally unrelated, or are independent. For continuous random variables, the differential entropy and mutual information are defined as follows:

(4) $$I\left(X,Y\right)\;=-\int p\left(x,y\right)\log_{2}\frac{p\left(x,y\right)}{p\left(x\right)p\left(y\right)}dxdy$$

However, the attributes selected based on the maximum dependency relationship may be redundant; that is, the new candidate features may be related to some previously selected features. In addition, it is known that if two attributes are highly correlated, and one of the attribute is deleted, the corresponding category discrimination ability will not change drastically. Accordingly, the redundancy between attributes should be fully considered in the process of multi-label attribute selection. This differs from traditional single-label attribute selection in that multi-label attribute selection involves not only redundancy between attributes, but also pairwise dependencies between attributes of each class of labels that need to be considered.

Based on our previous study [45], the membership of an object *x* ∈ *U* (*U* denotes a non-empty set with finite objects) in the fuzzy positive region is represented as *POS_B_*(*D*)(*x*). With the definition of the fuzzy positive region, the fuzzy dependency function can be computed by using the following formula:

(5) $$\gamma_{B}(D)=\frac{\sum_{x\in U}POS_{B}(D)(x)}{\left|U\right|}$$

If $\gamma_{B}(D)$ = 0, then the set of decision features *D* is independent of the set of condition features *B*. If $\gamma_{B}(D)$ = 1, then the set of decision features *D* depends completely on the set of condition features *B*. If 0 < $\gamma_{B}(D)$ < 1, then the set of decision features *D* depends partially on the set of condition features *B*.

Firstly, the framework we proposed establishes a multi-label representation model from the completed courses. For example, course 1 contains several crucial information features, such as the number of hours, credits, attendance, and mid-term and final grades. The second step is the attribute reduction process. Due to the large amount of feature information, some features may be weakly correlated with the predicted label, or have high repetitive feature importance. Therefore, after attribute reduction, features with more attribute representation ability and predictive value are retained. The last step is to perform multi-label prediction.

In the multi-objective optimization objective function, the previously mentioned mutual information and fuzzy dependency are applied to assess the independence and dependency among attributes, and between attributes and labels, respectively. Given that an instance *x* belongs to a group of training examples with values of labels, the attribute Fi is a discriminative feature, $\gamma_{Fi}^{L_{k}}\left(x\right)$ is the distinguishing ability of the attribute Fi with respect to the label *L*, and the quality of the attribute Fi is defined as:

(6) $$FD\left(Fi,L\right)\;=\;\left\{\begin{array}{c} \frac{I\left(Fi:L\right)}{1\;-\;\gamma_{Fi}^{L}\left(x\right)},\;\;\;\gamma_{Fi}^{L}\left(x\right)\;\neq 1\\ +\infty ,\;\;\;\;\;\;\;\;\;\;\;\;\;\;\;\;else \end{array}\right.$$

where $Z\left(Fi,L\right)$ denotes the ability of Fi to discriminate between labels *L*. A larger value of FD indicates a stronger feature discrimination capacity.

**Figure 2:**
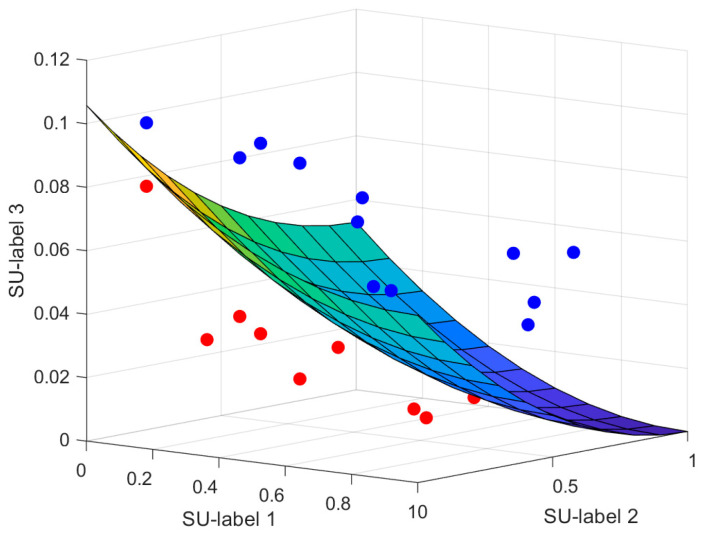
An instance of a Pareto optimal frontier for a multi-label dataset with 20 attributes and 3 labels.

In the multi-label dataset, calculate the symmetric uncertainty among each attribute and each label to construct the matrix *FD*, as follows [46]:

(7) $$FD=\left[\begin{array}{cccc} FD_{f_{1},l_{1}} & FD_{f_{2},l_{1}} & \ldots & FD_{f_{n},l_{1}}\\ FD_{f_{1},l_{2}} & FD_{f_{2},l_{2}} & \ldots & FD_{f_{n},l_{2}}\\ \vdots & \vdots & \ddots & \vdots \\ FD_{f_{1},l_{m}} & FD_{f_{2},l_{m}} & \ldots & FD_{f_{n},l_{m}} \end{array}\right]$$

where *FD*(*i*,*j*) shows the distinction between the *i*-th label and the *j*-th attribute. To follow up, construct an m-dimensional space, and expand features in these spaces based on the value of the matrix. Figure 2 shows the characteristics of a multi-label dataset with 3 labels in the construction space (*m* = 3).

Circles represent elements, from which we select the most prominent one. As mentioned earlier, it is suggested to find and select those features that belong to the Pareto optimal set as the final features in the multi-objective issues, as well as deleting other features. In Figure 2, the blue circles correspond to the non-dominant attributes, consisting of the Pareto optimal subsets and the neighboring dominant attributes (red circles). According to the legend, each point has a larger *FD* compared to the other two points, and has the largest value in at least one dimension; therefore, these attributes are not preferred to one another, and should not be removed. In addition, Figure 2 presents a scenario in which a red circle is associated with at least one blue circle that possesses a larger *FD* value in all dimensions; in short, the blue circle contains more specific features than the red circle.

The red circles (features) and blue circles (features) are redundant, and since the blue features have a higher *FD* value, they have more information that leads to the elimination of the red features. For a better understanding, a Pareto optimal front, resembling a spherical surface, is drawn, which separates the dominant and non-dominant features.

The red circles indicate dominant attributes, while the blue circles indicate non-dominant features. Compared with other recent multi-label feature selection approaches, this is a fast and accurate method. As mentioned in the first section, although there is a very limited number of works that use multi-objective concepts for multi-label feature selection [47,48], this method is unlike them in that it is intended for multi-label issues. Moreover, this method can be easily used for incremental attribute selection, which means that the new instances and attributes will arrive in sequence [49].

**Figure 3:**
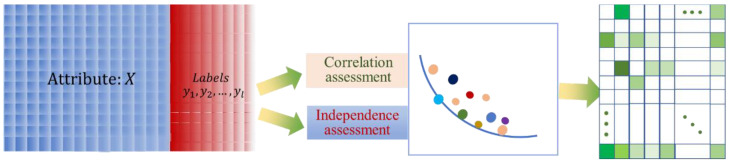
Flowchart of the multi-label attribute selection method.

Figure 3 shows the flowchart of the multi-label attribute selection method. Pre-course student performance data with multiple attributes and labels are used as the input matrix, and then the attributes and labels are evaluated simultaneously with correlations and dependencies based on the Pareto frontier multi-objective optimization method, resulting in attributes of high importance, as shown in the right-hand matrix, wherein the columns with more colored squares represent the attributes of high importance.

## 4. Results

In this section, we will empirically evaluate the proposed method with several of the latest multi-label attribute selection algorithms. To begin with, the features and comparison methods of multi-label datasets are introduced, followed by reporting of the performance of the AMuL through graphs and tables. Subsequently, we analyze and discuss the experimental results.

### 4.1. Data Preparation

This experiment was based on a dataset collected by higher education institutions. The dataset contains a total of more than 1000 students in 10 majors. For example, CEE comprises the three majors of the School of Mechanical and Electrical Engineering, instances represent the number of students in the major, and features represent the characteristics of each course of the major. For example, the electrical and electronic technology courses include the number of teaching hours (theoretical and experimental hours), attendance rate, mid-term and final grades, training grades, and other characteristics. Labels indicate the label of the course to be taken in the future semester, which is to predict the grade of the student in the upcoming course, along with their chance of “failure”. In addition, 85% of all samples are used for training, and 15% for testing.

### 4.2. Evaluation Indicators

As described in the previous section, we regard predicting the performance of students in each major as a task, and each task has multiple courses to predict. Compared with traditional supervised learning, the performance evaluation function of multi-label learning is somewhat complicated, because each instance belongs to a set of labels simultaneously. In this study, we evaluate each algorithm based on the average performance of all target courses [50].

Given a set of *n* multi-label training instances, $D=\;\left\{\left(x_{i},Y_{i}\right)|1\leq i\leq n\right\}$, where $x_{i}$ is described by a d-dimensional feature vector $F=\;\left\{F_{1,}F_{2},\ldots ,F_{d},\right\}$, which means that $Y_{i}=\;\left\{L_{1},L_{2},\ldots ,L_{k}\right\}$ is a sequence of labels associated with $x_{i}$ that are presented in sequential order. The task of multi-label feature selection is to select a compact feature subset from F without decreasing the predictive classification performance. To evaluate the performance of multi-label feature selection algorithms, we select average precision, ranking loss, Hamming loss, coverage, and one-error as evaluation metrics [40]. Given a test set $T=\;\left\{\left(x_{i},Y_{i}\right)|1\leq i\leq m\right\}$, and the family of *q* learned functions, $f=\;\left\{f_{1,}f_{2},\ldots ,f_{q},\right\}$.

Average precision (AP) evaluates the average proportion of relevant labels ranked higher than a particular label $L_{k}\in Y_{i}$.

(8) $$AP=\frac{1}{m}\sum_{i=1}^{m}\frac{1}{\left|Y_{i}\right|}\sum_{L_{k}\in Y_{i}}\frac{\left|\left\{L_{k}\in Y_{i}:r\left(x_{i},L_{j}\right)\;\leq r\left(x_{i},L_{k}\right)\right\}\right|}{r\left(x_{i},L_{k}\right)}$$

The larger the value of the *AP* metric, the higher the performance.

Ranking loss (*RL*) evaluates the average proportion of reversely ordered label pairs.

(9) $$RL=\frac{1}{m}\overset{m}{\underset{i=1}{\sum }}\frac{1}{|Y_{i}|\left|\tilde{Y_{i}}\right|}\left|\left\{\left(L_{k},L_{j}\right)|f_{k}\left(x_{i}\right)\;\leq f_{j}\left(x_{i}\right),\left(L_{k},L_{j}\right)\;\in Y_{i}\times \tilde{Y_{i}}\right\}\right|$$

where $\tilde{Y_{i}}$ means the complementary set of label space *L* on a given instance $x_{i}$. The smaller value of RL indicates the better performance of the method.

Hamming loss (*HL*) evaluates the proportion of misclassified example–label pairs.

(10) $$HL=\frac{1}{m}\overset{m}{\underset{i=1}{\sum }}\frac{\left|p\left(x_{i}\right)⊕Y_{i}\right|}{k}$$

where ⊕ means the symmetric difference between the true label set $Y_{i}$ and the predicted label set $p\left(x_{i}\right)$. Here, the smaller the value of HL, the better the performance of the method.

Coverage (*CV*) evaluates the average distance we need to go down the list of labels in order to cover all of the appropriate labels of a given sample $x_{i}$.

(11) $$CV=\frac{1}{q}(\frac{1}{m}\overset{m}{\underset{i=1}{\sum }}\underset{L_{k}\in Y_{i}}{\max}\;r\left(x_{i},L_{k}\right)\;-\;1)$$

where the definition of $r\left(x_{i},L_{k}\right)$ = $\;\frac{1}{q}\left[\left[f_{k}\left(x_{i}\right)\;\leq f_{j}\left(x_{i}\right)\right]\right]$ is consistent with the above definition of $r\left(x_{i},L_{k}\right)\;$ in *AP*. A smaller value of *CV* indicates better performance of the method.

One-error (*OE*) calculates the number of occurrences in which the top ranked label is not in the appropriate multi-label set of a sample $x_{i}$.

(12) $$OE=\frac{1}{m}\overset{m}{\underset{i=1}{\sum }}⟦[arg\;\underset{L_{k}\in L}{\max}\;f\left(x_{i},L_{k}\right)]\;\notin Y_{i}⟧$$

A smaller value of OE indicates better performance of the method. In particular, if OE=0, the performance of the method is perfect.

For these evaluation metrics, Hamming loss focuses on evaluating the label set prediction performance for each instance, while the other four evaluation metrics are more concerned with the performance of label ranking.

### 4.3. Experimental Results

**Figure 4:**
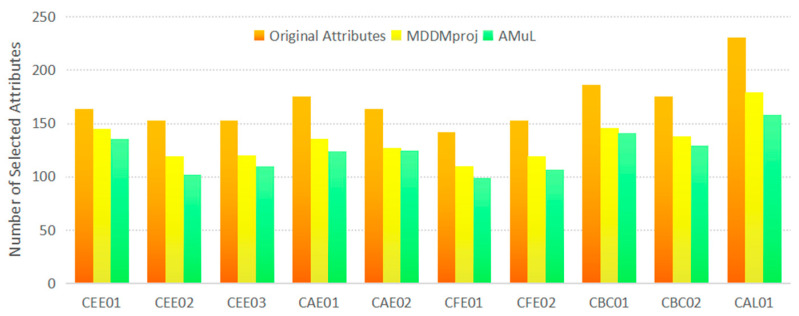
The number of selected features (The labels of the x-axis from 1 to 10 denote the 10 datasets described in Table 2.

In order to prove the effectiveness of AMuL, we compare our algorithm with MLNB [51], MDDMproj [52], MLFRS [53], MFNMI [54], RF-ML [55], and AMI [56]. Figure 4 shows the comparison of the number of selected features in the original attributes, MDDMproj, and AMuL. It can be seen that, compared with the number of original attributes, MDDMproj and AMuL obtained a more streamlined feature set through attribute reduction. On average, our proposed AMuL method has a higher reduction rate than MDDMproj in most professional data samples. Thus, a question emerges as to whether it possesses the same predictive accuracy on a more condensed feature set. For the sake of fairness, we used the reduced feature set of the above six comparison methods for classification. We then used a fivefold cross-validation method in the experiments to record the best results of the parameters in terms of classification performance. The different class groups k were set to 10 in order to prevent overfitting of the data, and to balance the complexity of the model according to the literature [57]. All methods were fully implemented in MATLAB and tested on a PC equipped with an 8-core 1.80 GHz Intel Core processor and 16 GB RAM.

Table 3, Table 4, Table 5, Table 6 and Table 7 show the predictive performance of the seven comparison algorithms in five metrics: *AP*, *RL*, *HL*, *CV*, and *OE*, respectively. The symbol ↑ indicates a larger value for better performance; the symbol ↓ indicates a smaller value for better performance. For the results of all methods under each dataset, special comments in bold are used for easy identification. The last row of each table summarizes the statistics of the cases in which the AMuL algorithm is better than (Win), equivalent to (Draw), or less than (Loss) the comparison algorithms in this performance evaluation.

In Table 3, it is safe to conclude the following: (1) Among the 10 major curriculum datasets, the proposed AMuL has eight superiorities to the comparison algorithms on average, and one equivalency to the comparison algorithms. (2) In terms of average prediction performance with different evaluation indicators, AMuL is significantly better than all comparison algorithms. (3) In addition, the count of victory/failure records shows that in terms of each evaluation index, AMuL is more competitive than the six comparison algorithms. For example, in the AP evaluation index, the AMuL algorithm achieved four wins, one draw, and one loss in a contest with six comparison algorithms.

The above-mentioned experiment was dedicated to dealing with the problem of predicting the performance of students in the pre-class setting, attempting to use students’ learning in previous courses to predict their risk of failing in the new semester’s relevant advanced course, so as to assist teachers or administrators in teaching, studying, and research in accordance with their aptitude in the early stages of the course. Compared with six advanced methods, the proposed AMuL obtains competitive prediction performance and improves predictability. Traditional modeling work is often carried out during the course in question, or even near to its end. Therefore, most of the “risky students” detected by the model have already displayed poor learning performance on the course (risk has already occurred).

At this time, the academic warning is issued. Even if the student can pass the course exam, they still lack the overall mastery of the relevant background and pre-knowledge. The method proposed in this article can be modeled before the start of the course, and the detected “risk students” are only “potential risks” (risks have not yet occurred). If the teachers at the beginning of the course give extra attention and guidance to risky students, they can not only avert the transformation of “potential risks” into “actual risks”, but can also cultivate students’ interest in learning and enhance their confidence in the curriculum, thereby improving students’ overall mastery of the curriculum.

In addition, by transforming the pre-class student performance prediction problem into a multi-label processing issue, the AMuL method fully integrates the actual situation of pre-class student performance prediction, which is more in line with the prediction requirements. In terms of the modeling method, considering that there is a certain correlation between multiple target courses, as well as between target courses and pre-order courses, a multi-label attribute reduction algorithm is introduced to improve the expressive ability of features. In the 10 different professional student courses of the dataset, the effectiveness of this method was verified.

Although the proposed method achieved significant predictability and accuracy in predicting student performance, it is constrained by the limited sample data information, and the generality of the method needs to be tested on more datasets—especially in light of the gradual development of smart campuses, via which the video, image, and voice data related to students’ in-class learning behaviors can be effectively collected. In addition, with the rapid development of online course tools, a number of digital tools [14] and online platforms [37] are increasingly being deployed in educational scenarios, and the educational data generated from these educational venues still merit extended research using the approach proposed in this study. With the help of these richer teaching resources, the multi-label attribute selection method can be combined with more advanced technologies and methods. For example, it can combine computer vision, natural language processing, and other algorithms for student performance prediction.

## 5. Discussion and Conclusions

This article targets traditional classroom teaching scenarios in colleges and universities, and is committed to improving the predictability and accuracy of the method by mining the coupling relationship between the completed course scores and subsequent courses. (1) For the first time, this article proposes the use of multi-label attribute selection methods to streamline the data characteristics of the students’ courses and improve the expression ability of the original feature set. (2) Aiming at the problem of predictive lag in existing research, through the “pre-class” student performance prediction method, students’ previous semester learning conditions in each course can be used to predict their risk of failing in subsequent courses. The multi-label method is more in line with the characteristics of entry-level courses, and can directly deal with multi-course prediction problems. (3) The method in this paper does not rely on the process information of the course to be carried out, but works directly through the student’s performance history on previous courses, which can relatively accurately predict the student’s performance before the course starts, and has better predictability and practicality. This method was verified on real datasets of 10 different professional courses.

## Figures and Tables

**Table 1:** Related studies for student performance prediction task.

Authors	Year	Features
Macfadyen and Dawson [29]	2010	Predictive modeling of students’ final grades using factors such as student discussion information, number of emails sent, and test completion.
Zafra et al. [30]	2011	Use of information such as quizzes, assignments, forums, etc., to predict whether a student will pass or fail the course.
Sweeny et al. [4]	2015	Predicting grades for the next semester based on information about students’ grades in completed courses.
Ren et al. [25]	2016	Applying data from MOOC server logs to predict learning outcomes.
Conijn et al. [36]	2018	Predicting student performance and discovering the potential for MOOC improvements.
Oswaldo et al. [37]	2019	Comparing different educational data mining (EDM) algorithms to discover research trends and patterns in graduation rate indicators.
Ma et al. [22]	2020	Multi-instance multi-label learning for pre-course student performance prediction.
Ma et al. [36]	2020	Multi-instance multi-label learning with multi-task learning for pre-course student performance prediction.

**Table 2:** Characteristics of multi-label datasets.

Data Sets	Instances	Features	Labels	Train	Test
CEE01	102	164	4	87	15
CEE02	58	153	3	49	9
CEE03	64	153	3	54	10
CAE01	83	175	5	71	12
CAE02	61	164	4	52	9
CFE01	205	142	4	174	31
CFE02	137	153	5	116	21
CBC01	92	186	7	78	14
CBC02	86	175	6	73	13
CAL01	317	231	10	269	48

**Table 3:** Predictive performance of each comparison algorithm in terms of average precision (↑).

Datasets	AMI [56]	RF-ML [55]	MFNMI [54]	MDDMproj [52]	MLFRS [53]	MLNB [51]	AMuL
CEE01	0.81	0.81	0.81	0.80	0.81	0.81	0.81
CEE02	0.84	0.78	0.83	0.81	0.80	0.83	0.84
CEE03	0.78	0.78	0.79	0.77	0.80	0.74	0.80
CAE01	0.75	0.75	0.74	0.51	0.74	0.75	0.75
CAE02	0.75	0.76	0.74	0.75	0.75	0.77	0.77
CFE01	0.61	0.99	0.83	0.61	0.85	0.69	0.89
CFE02	0.80	0.80	0.80	0.80	0.81	0.81	0.81
CBC01	0.88	0.89	0.88	0.85	0.89	0.89	0.89
CBC02	0.85	0.86	0.87	0.86	0.86	0.82	0.88
CAL01	0.76	0.73	0.75	0.78	0.81	0.80	0.80
Win/Draw/Loss	10/0/0	10/0/0	10/0/0	10/0/0	9/0/1	9/1/0	-

**Table 4:** Predictive performance of each comparison algorithm in terms of ranking loss (↓).

Datasets	AMI [56]	RF-ML [55]	MFNMI [54]	MDDMproj [52]	MLFRS [53]	MLNB [51]	AMuL
CEE01	0.17	0.17	0.17	0.17	0.17	0.17	0.17
CEE02	0.16	0.21	0.17	0.19	0.19	0.20	0.16
CEE03	0.23	0.24	0.23	0.25	0.23	0.23	0.23
CAE01	0.16	0.14	0.12	0.12	0.10	0.10	0.10
CAE02	0.17	0.16	0.16	0.16	0.17	0.16	0.16
CFE01	0.08	0.07	0.07	0.07	0.07	0.07	0.07
CFE02	0.07	0.07	0.07	0.08	0.07	0.07	0.07
CBC01	0.13	0.13	0.14	0.14	0.13	0.13	0.10
CBC02	0.11	0.10	0.10	0.10	0.11	0.12	0.10
CAL01	0.17	0.18	0.17	0.16	0.17	0.17	0.16
Win/Draw/Loss	10/0/0	10/0/0	10/0/0	10/0/0	9/0/1	9/1/0	-

**Table 5:** Predictive performance of each comparison algorithm in terms of Hamming loss (↓).

Datasets	AMI [56]	RF-ML [55]	MFNMI [54]	MDDMproj [52]	MLFRS [53]	MLNB [51]	AMuL
CEE01	0.072	0.069	0.065	0.064	0.061	0.057	0.057
CEE02	0.060	0.064	0.066	0.067	0.059	0.063	0.057
CEE03	0.055	0.060	0.052	0.062	0.064	0.058	0.052
CAE01	0.075	0.072	0.078	0.064	0.076	0.074	0.066
CAE02	0.083	0.083	0.087	0.086	0.078	0.079	0.078
CFE01	0.044	0.045	0.048	0.048	0.056	0.043	0.050
CFE02	0.049	0.054	0.052	0.047	0.058	0.049	0.046
CBC01	0.036	0.026	0.031	0.035	0.033	0.029	0.028
CBC02	0.041	0.045	0.035	0.047	0.035	0.040	0.034
CAL01	0.088	0.074	0.081	0.070	0.076	0.082	0.070
Win/Draw/Loss	10/0/0	9/0/1	10/0/0	9/0/1	10/0/0	08/1/1	-

**Table 6:** Predictive performance of each comparison algorithm in terms of coverage (↓).

Datasets	AMI [56]	RF-ML [55]	MFNMI [54]	MDDMproj [52]	MLFRS [53]	MLNB [51]	AMuL
CEE01	3.86	3.78	3.84	3.83	3.83	3.82	3.75
CEE02	4.61	4.51	3.83	4.10	4.11	4.33	3.55
CEE03	5.08	5.26	4.95	5.41	5.25	5.05	4.95
CAE01	3.65	3.18	3.55	3.71	3.46	3.06	2.78
CAE02	3.50	3.56	3.52	3.64	3.74	3.52	3.50
CFE01	3.09	3.04	3.11	3.10	3.08	2.93	2.93
CFE02	2.53	2.47	2.46	2.50	2.51	2.70	2.42
CBC01	1.85	1.82	1.88	1.95	1.84	1.82	1.81
CBC02	1.88	1.86	1.87	1.84	1.82	1.85	1.79
CAL01	3.79	3.94	3.80	3.56	3.76	3.58	3.25
Win/Draw/Loss	10/0/0	10/0/0	9/0/1	10/0/0	10/0/0	9/1/0	-

**Table 7:** Predictive performance of each comparison algorithm in terms of one-error (↓).

Datasets	AMI [56]	RF-ML [55]	MFNMI [54]	MDDMproj [52]	MLFRS [53]	MLNB [51]	AMuL
CEE01	0.36	0.34	0.33	0.32	0.30	0.29	0.28
CEE02	0.30	0.32	0.33	0.33	0.30	0.32	0.29
CEE03	0.27	0.30	0.26	0.31	0.32	0.29	0.26
CAE01	0.38	0.36	0.39	0.32	0.38	0.37	0.33
CAE02	0.41	0.42	0.44	0.43	0.39	0.38	0.39
CFE01	0.22	0.23	0.24	0.24	0.28	0.22	0.25
CFE02	0.25	0.27	0.26	0.24	0.29	0.25	0.23
CBC01	0.18	0.13	0.15	0.17	0.17	0.15	0.19
CBC02	0.20	0.22	0.18	0.24	0.18	0.20	0.17
CAL01	0.44	0.27	0.41	0.35	0.38	0.41	0.35
Win/Draw/Loss	10/0/0	9/0/1	9/1/0	10/0/0	10/0/0	9/0/1	-

## Data Availability

The data are not publicly available due to restrictions on the data by the Registrar’s Office of the College.
